# Twenty-Seven Years After Sex Reassignment Surgery in Female Transgender Patients: Is Prolapse of the Neovagina an Issue?

**DOI:** 10.1007/s00192-025-06251-6

**Published:** 2025-08-02

**Authors:** Ramona Osswald, Anna-Sophie Villiger, Giovanni Ruggeri, Diana Hoehn, Michael Mueller, Annette Kuhn

**Affiliations:** 1 Department of Obstetrics and Gynecology, Cantonal Hospital of Schaffhausen, Geissbergstrasse 81, 8208 Schaffhausen, Switzerland; 2 Department of Obstetrics and Gynecology, Bern University Hospital and University of Bern, Friedbühlstrasse 19, 3010 Bern, Switzerland

**Keywords:** Male-to-female transgenders, Sex reassignment surgery, Vaginal prolapse, Rectocele, POP-Q score

## Abstract

**Introduction and Hypothesis:**

Various techniques for neovagina creation have been developed and refined. The aim of this study was to evaluate the incidence of prolapse and possible consecutive therapies in transfemale patients who have received a neovagina as part of their sex reassignment surgery (SRS).

**Methods:**

This prospective single-centre case control study was performed at Bern University Hospital (Department of Gynaecology) between 2017 and 2023. Sixty-eight patients who had received SRS (all male to female) were undergoing regular gynaecologic examinations assessing the ICS-Pelvic Organ Prolapse Staging (POP-Q score) and VAS score for symptom burden. Appropriate management for prolapse correction was initiated. Linear and logistic regression were employed for the average comparison of the parameters in correlation to the type of sex reassignment surgery applied.

**Results:**

Fifty-four of the 68 participant patients had undergone penis–scrotum inversion technique, six had a neovagina created by peritoneum and seven had an intestinal neovagina. Mean follow-up was 27.5 years. Thirteen patients (19.4%) experienced genital prolapse in this cohort. The group after peritoneal neovagina surgery demonstrated the highest odds ratio for rectocele (OR 4.9, *p* = 0.17 95% CI 0.71–33.78) and vaginal prolapse (OR 16.67, *p* = 0.005 95% CI 2.3–120.65). Statistically significant differences in all POP-Q parameters for the penile inversion group indicate smaller vaginal prolapse. Prolapse surgery significantly decreased the VAS score (*p* < 0.001; 95% CI 5.92–8.38).

**Conclusions:**

One in five transfemale patients who have undergone sex reassignment surgery experience genital prolapse. The prevalence of prolapse was found to be highest in the peritoneum reconstruction group, followed by the patients with intestinal neovagina and penile inversion. Surgical intervention for prolapse appears to significantly alleviate symptoms.

## Introduction

In recent decades, there has been evidence of a growing reported transgender population. The overall prevalence for transsexualism in European countries is 6.8 for trans women and 2.6 for trans men in 100,000 individuals [1–4], while the prevalence for male-to-female gender dysphoria is between 5 and 14 per 1000 adult male individuals (0.015–0.014) and 2 and 3 per 1000 adult female individuals (0.002–0.003) for female-to-male gender dysphoria in the USA according to DSM-559 [2]. Patients considering sex reassignment surgery (SRS) are facing life-changing and irreversible consequences that are only becoming apparent several decades after the first techniques have been established [4–6]. In the transfemale population, one of these potential conditions is genital prolapse. There is a paucity of long-term data analysing the incidence of neovagina prolapse in the transfemale population. As for the creation of neovaginae, various techniques are available. The penile skin inversion technique was first described in 1957 [5, 6] and has since become the most frequently applied surgical method for male-to-female sex reassignment surgery (approximately 85%) [7]. This technique involves the utilisation of penile and scrotal skin as flaps or grafts to form the neovagina. Alternatively, the technique of sigmoidocolpoplasty involves the transplantation of a pedunculated sigmoidal flap to create the neovagina [8, 9]. Other intestinal grafts may be employed. The utilisation of peritoneal flaps in the formation of the neovagina is a possible procedure. A number of techniques have been described, including the use of peritoneum from the posterior bladder wall and anterior sigmoid [10]. It is also frequently applied in cases of secondary repair of the neovagina or as a combination method for penile vaginoplasty if the penile/scrotal skin is insufficient [7, 11]. The aim of this study is to assess the long-term incidence of prolapse in patients after man-to-female SRS, considering different surgical techniques, and to present the management of subsequent prolapse.

## Materials and Methods

This prospective quality control single-centre study was conducted at the gender clinic, Department of Obstetrics and Gynecology of the University Hospital of Bern. Sixty-eight patients who underwent sex reassignment surgery (all from male to female) were undergoing regular yearly gynaecological examinations between January 2017 und December 2023. The type of SRS was verified according to patients’ reports and, in cases where available, operation reports.

In all of these patients, prolapse staging was performed using the ICS-Pelvic Organ Prolapse Staging (POP-Q) score [4, 12]. The ICS/POP-Q staging system utilises a score comprising nine parameters to ascertain the position of the anterior, posterior and apical vaginal segments. All examinations were carried out in the lithotomy position with no prior emptying of the bladder by the same examiner (A.K.). A speculum was used consistently. Patients were asked to perform either a Valsalva manoeuvre or cough. Additionally, demographic data, age at SRS, the exact SRS technique, body mass index, residual urine, prolapse symptoms and potential prolapse therapies were noted.

If prolapse developed, patients were asked about the bother they experienced by prolapse applying the visual analogue scale (VAS) with a rating from 0 = no bother to 10 = most imaginable bother. The exact wording was “How much are you bothered by the prolapse using this scale from 0–10 with 10 being the greatest imaginable bother?” Depending on the general fitness of the patient and the wish to maintain vaginal intercourse or not the choice of consecutive conservative and/or surgical therapies was discussed with the patients. If surgery was indicated, VAS was used in the same way at the 6 weeks postoperative follow-up.

Statistical analysis was conducted utilizing Stata 16 (Stata Corporation, College Station, TX). The median, range, mean and standard deviation for continuous variables were calculated, while the percentages for the qualitative variables were determined. Linear and logistic regression were employed for the average comparison. Cases with missing values were excluded. Statistical significance was determined as *p* < 0.05.

Approval by the IRB/ethics committee was waived for this quality control single-centre study, which described our standard care in accordance to current Swiss legislation. Prior to the examination and therapy, all patients provided informed consent for their clinical data to be utilized in research studies, with strict confidentiality being maintained. This study adheres to the Strengthening the Reporting of Observational Studies in Epidemiology (STROBE) statement [13].

## Results

**Fig. 1:**
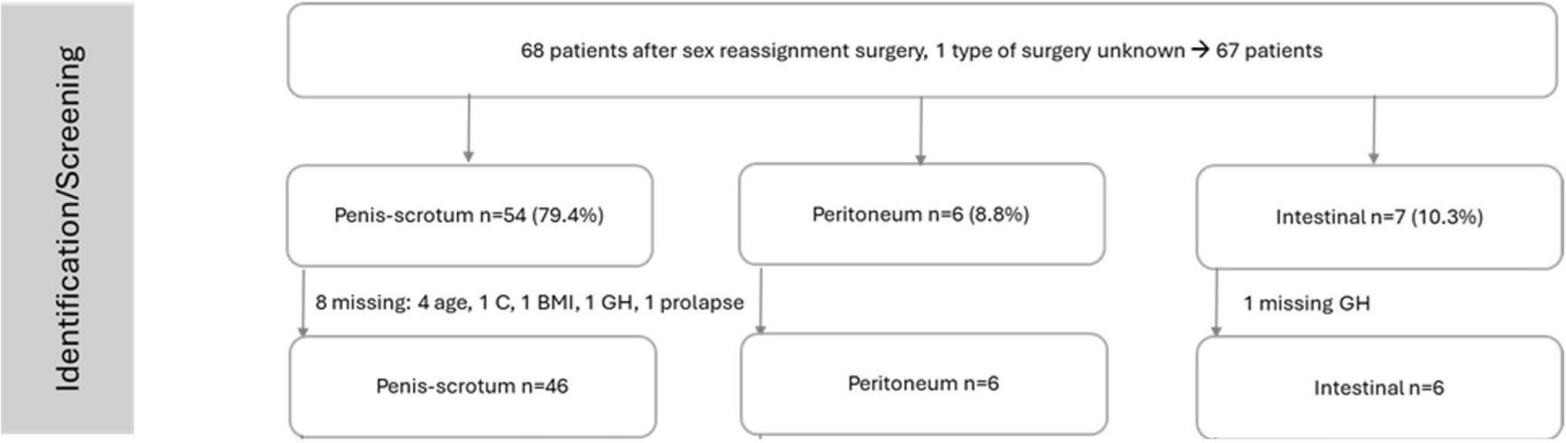
Flowchart collective, type of prolapse

A total of 68 transfemale patients were included in the study. Table 1 shows demographic data and type of SRS. Median follow-up time since the SRS was 27.5 years (range 16–52). Fifty-four (79.4%) patients had undergone penis–scrotum reconstruction involving either one scrotal procedure or 53 penile/scrotal inversions. Six (8.8%) patients had undergone peritoneal vaginoplasty in the past, while seven (10.3%) had received intestinal vaginoplasty whereof five involving sigma, one involving ileum and one involving sigma or ileum (Fig. 1). The patients underwent the procedure in a variety of medical centres located in Switzerland, the United Kingdom, Bulgaria, Germany, the United States, and Thailand.

**Table 1:** Demographics

	*n* = 68	Missing
Age at SRS OP, years, median (range)	43 (19–78)	4
Type of surgery for Neovagina, *n* (%)		1
Penis–scrotum	54 (79.41)	
Peritoneum	6 (8.82)	
Intestine	7 (10.29)	
Follow-up, years, median (range)	27.5 (16–59)	0
BMI, kg/m^2^, median (range)	28 (19–41)	1

*N* number, *SRS* Sex reassignment surgery, *BMI* Body mass index

Table 2 shows results of POP-Q scores in median and range, type of prolapse, residual urine and other pathological findings. Until December 2023, a total of 13 patients had been documented to have experienced symptomatic or asymptomatic prolapse of the neovagina, cystocele or rectocele. Of these, seven cases were classified as rectocele, while seven cases exhibited vaginal prolapse (one patient with both rectocele and vaginal prolapse). The prevalence of genital prolapse was found to be 17.4% in the penile inversion group, 83% in the peritoneum reconstruction group, and 14.3% in the intestinal group. Of interest, no significant and/or symptomatic cystocele could be noted. Two patients developed stricture of the urethra, which required repeated operative opening, but still caused significant residual urine, so the patients are still practising intermittent self-catheterization. In both cases, the neovagina was formed by penis–scrotum inversion.

**Table 2:** Prolapse and other pathological findings

	*n* = 68	Missing
POP-Q score, cm, median (range)		
Aa	*−2 (−3 to 1)	0
Ba	*−2 (−2.5 to 4)	0
C	*−9 (−15 to 12)	1
TVL	9 (2 to 17)	0
Ap	*−2 (−3 to 2.5)	0
Bp	*−2 (−2 to3)	0
PB	5 (3–9)	0
GH	4 (2–7)	2
Rectocele, *n* (%)	7 (10.29)	0
I	3 (4.41)	
II	1 (1.47)	
III	3 (4.41)	
Vaginal prolapse, *n* (%)	7 (10.29)	1
II	3 (4.41)	
III	4 (5.88)	
Pelvic organ prolapse, *n* (%)	13 (19.17)	1
Residual urine, ml, median (range)	32.5 (0–330)	0
Urethral stricture, *n* (%)	2 (2.94)	0

*N * number, *POP-Q score* Pelvic Organ Prolapse Quantification score, *Aa/Ba* anterior wall, *C* cervix or cuff, *GH* genital hiatus, *PB* perineal body, *TVL* total vaginal length, *Ap/Bp* posterior wall

Overall, nine patients were excluded from the final analysis due to missing data. For one patient, the type of sex reassignment surgery was unknown. Post-penis–scrotum surgery, four patients had missing age data, one had a missing C parameter, one had a missing BMI, and one had a missing GH parameter. In another patient in the intestinal (ileal) vaginoplasty group, the GH parameter was also missing. These nine patients were excluded from the subsequent analysis (they all had no genital prolapse). Another patient who underwent penis–scrotum vaginoplasty and for whom the documentation of vaginal prolapse (yes/no) was absent was excluded from the study, resulting in the final inclusion of 58 patients. Of these 58 cases, 46 had undergone penis–scrotum reconstruction, six intestinal reconstruction and six peritoneal reconstruction.

Fourteen patients received further therapy after sex reassignment surgery for either prolapse or urethral stricture (Table 3). Five patients received a pessary, three had to perform regular intermittent self-catheterisation and 11 (78.6%) of them underwent surgery. Of the 11 operations,two were colporrhaphia posterior, one was a laparoscopic rectopexy, three were laparoscopic sacrocolpopexy, four were colpectomies and two were meatotomies. A significant reduction in the VAS score was observed in the post-operative period when compared to the pre-operative condition. In the penile inversion cohort, eight patients were identified as having genital prolapse, of whom a total of five patients underwent surgical intervention. The remaining three patients were treated with pessaries or did not express a desire for additional treatment. Conversely, all patients with genital prolapse in the intestinal and peritoneal reconstruction group underwent prolapse surgery.

**Table 3:** Therapy

	*n* = 14	
Pessary	5 (35.71)	
ISK	3 (21.43))	
Surgery	11 (78.57)	
Colporrhaphia posterior, *n* (%)	2 (18.18)	
LSC rectopexy, *n* (%)	1 (9.09)	
LSC sacrocolpopexy, *n* (%)	3 (27.27)	
Colpectomy, *n* (%)	4 (36.36)	
Meatotomie	2	
VAS preoperative, mean (SD)	7.85 (1.77)	
VAS postoperative, mean (SD)	0.69 (0.86)	
VAS diff., mean (SD)	7.15 (2.04)	*p* < 0.001 (95% CI 5.92–8.38)

*N* number, *ISK* intermittent self-catheterization, *LSC* laparoscopic, *VAS* visual analog scale

A subgroup analysis was conducted to compare the odds ratio for rectoceles, vaginal prolapse and POP-Q parameters between the groups (Table 4). The odds ratio for prolapse was found to be highest in the group that underwent peritoneal neovagina surgery for rectocele (OR 4.9, *p* = 0.17, 95% CI 0.71–33.78) and vaginal prolapse (OR 16.67, *p* = 0.005, 95% CI 2.3–120.65). The odds ratio for rectoceles (0.1, *p* < 0.001 95% CI 0.04–0.26) when compared to the peritoneum subgroup and for vaginal prolapse (0.06, *p* < 0.01 95% CI 0.02–0.19) when compared to the peritoneum and the intestine subgroup was significantly lower in the penis–scrotum collective. Table 5 summarizes the characteristics of patients with and without prolapse in relation to surgical method, BMI, age and residual urine.

**Table 4:** Subgroup analysis

Type of surgery for neovagina, *n* (%)				
	Rectocele, OR	*p* value (95% CI)	Vaginal prolapse, OR	*p* value (95% CI)
Penis–scrotum	0,1*	< 0.001 (0.04–0.26)	0.06*	< 0.001 (0.02–0.19)
Peritoneum	4.9	0.17 (0.71–33.78)	16.67	0.005 (2.3–120.65)
Interstine	–	-	2.78	0.41 (0.25–31.13)

**Table 5:** Subgroups cases and controls

	Prolapse	No prolapse
	*n* = 13	*n* = 55, 1 missing
Age at GA OP, years, median (range)	44 (31–67)	42 (19–78)
Type of surgery for neovagina, *n* (%)		
Penis–scrotum	8 (61,54)	46 (85.19)
Peritoneum	4 (30,77)	1 (1.85)
Intestine	1 (7.69)	6 (11,11)
Residual urine, ml, median (range)	30 (0–280)	35 (0–330)
BMI, median, range	31 (26–41)	27 (19–36)

*N* number, *SRS* Sex reassignment surgery, *BMI* Body mass index

**Fig. 2:**
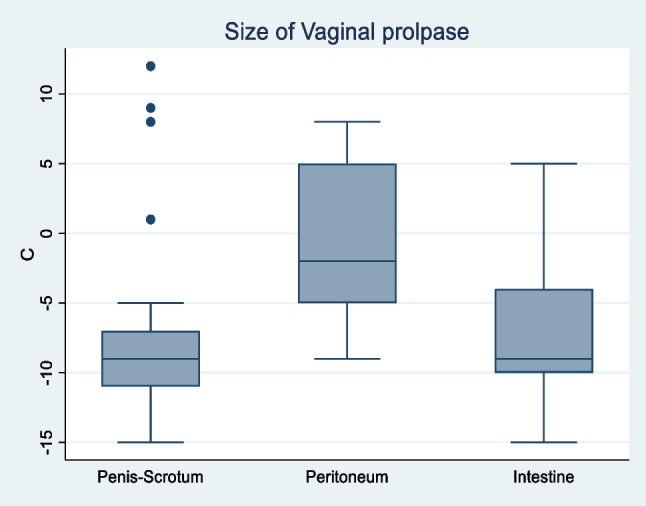
Boxplot size of vaginal prolapse

**Fig. 3:**
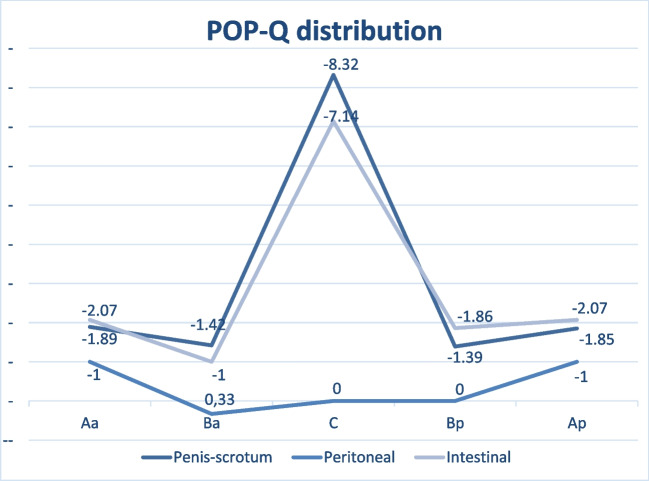
POP-Q distribution

As for the exact classification of the POP-Q score, a statistically significant difference was observed between the penis–scrotum subgroup and the other two groups in all POP-Q parameters. Figure 2 shows the size of the vaginal prolapse depending on the type of sex reassignment surgery applied; Fig. 3 provides an overview over the POP-Q distribution of the parameters Aa, Ba, C, Bp and Ba (mean of each parameter) in the different collectives. The vaginal apex C was found to be significantly higher in the penis–scrotum group when compared to the peritoneum and intestine groups (mean −8.32, *p* < 0.001, 95% CI −9.84- −6.8), resulting in a mean total vaginal length of 9.8 (*p* < 0.001, 95% CI 8.99–10.6). The size of the genital hiatus (mean 4.08, *p* < 0.001, 95% CI 3.79–4.36) and the perineal body (mean 5.15, *p* < 0.001, 95% CI 4.88–5.41) in the penis–scrotum collective were also significantly different when compared to the peritoneum and intestine surgery group.

## Discussion

This study demonstrates that neovaginal prolapse is a relevant long-term complication in transfemale patients decades after SRS. The data presented in this study indicates that patients should be informed about the possibility of prolapse in later life preoperatively. The majority of patients undergo SRS at a younger age (median age 37.7 years) [14], and therefore most of them do not experience genital prolapse at that time. The lower prevalence observed in the transgender population compared to the cis population (lifetime risk for prolapse 30−50% [15, 16]) may be attributable to the fact that all are nonparous and have a different anatomy of the pelvis and the pelvic floor. However, ageing is undoubtedly a risk factor for these patients. The highest prolapse rates were observed in the peritoneal group, suggesting a potential link between surgical technique and long-term anatomical outcomes.

It is imperative to emphasise that the occurrence of genital prolapse in trans women following SRS must be distinguished from that observed in cis women, since vaginoplasty for SRS exerts minimal impact on the pelvic suspension of the rectum or the bladder [7, 17]. In the case of male-to-female transgender individuals, genital prolapse is not typically attributed to a genuine loss of suspension of the bladder or rectum, but rather to a deficiency in the suspension of the graft used [14]. It is noteworthy that no isolated cystoceles were identified in either group, despite the similarity in Aa and Ap measurements and the presence of Ba and Bp. We hypothesize that the presence of the prostate may support the anterior compartment therefore preventing cystoceles, yet data concerning this topic in literature is missing. Apart from rare prolapse of the mucosa or the whole graft, stenosis of the distal vagina may occur with this technique due to shrinking scar tissue of the anastomosis [18, 19]. The size of the prolapse in the penile inversion group is significantly smaller than in the other two populations, especially when compared to the peritoneal reconstruction group. In addition, after penile inversion not all types and manifestations of prolapse are accompanied by bothersome symptoms, and in many cases, conservative or no treatment is required. However, patients requiring surgical treatment experienced symptom relief. For this reason, irrespective of the surgical technique initially employed, the operative treatment of genital prolapse in male-to-female transgenders must be adapted to suit each individual case.

Our study’s notable strength lies in use of validated tools to measure prolapse and patient reported outcomes. Another strength is the substantial number of cases operated across six different countries, with a prolonged follow-up period. At the same time, a weakness of the study is that—due to the long-time interval of the follow-up after SRS—we have no detailed operating notes from the SRS in all of the cases. We had to rely on the patients’ information concerning their SRS, which may be faulty. This inherits the potential risk of misclassification of surgical technique. Since the majority of patients were well informed about the type of surgery, we consider this weakness as minor. However, as patients were operated in many different international centres, the experience in SRS may have varied, possibly affecting the comparability of the surgeries performed which may influence the long-term outcome in respect of prolapse. In the interesting subject of data concerning prolapse after transfemale SRS, a total of 68 patients is a substantial number of cases. Nevertheless, the number may be still too small to draw a definitive conclusion.

In the systematic analysis of 24 studies comprising 3166 patients following different SRS since 1995, Tran et al. report a prevalence of prolapse of 2.5% with the penile inversion technique and 3.5% with the intestinal vaginoplasty. The only significant risk factors identified in the population were a high body mass index at the time of surgery [7] and the mean follow-up time was 22.5 months. Those numbers are lower than in the current study, but follow-up was much shorter so we may postulate that over time, prolapse risk is increasing. As the studies comprise pooled data of male-to-female transgenders and the postoperative course, the specific endpoint prolapse is not specified in most of the studies. Six studies (five penile skin, one intestinal reconstruction) report a 0% prolapse rate, apparently irrespective of fixation of the neovagina [7]. In addition, the systematic review encompassed a wide array of studies and populations, with outcomes and results that were largely noncomparable, as previously highlighted. For instance, in one of the studies in this systematic analysis, all patients who underwent peritoneal reconstruction had previously undergone other sex reassignment surgeries [7, 11].

The length of the neovagina in the penile inversion population is most similar to the TVL of women in the cis-population [20]: Schimpf et al. compared the vaginal length of 333 sexually active women to that of 172 women without regular sexual activity. The results of this study indicated that the vaginal length was slightly greater in the sexually active population (9.1 cm vs. 8.9 cm, *p* = 0.04) [20], while the size of the GH did not differ between the groups (3.2 cm vs. 3.1 cm, *p* = 0.58). Urethral stricture is the most common complication of penile inversion surgery, necessitating re-operation in approximately 14.4% of cases [18, 19]. The reconstruction of the urethra is a complex procedure involving the resection of the redundant spongiosum and the appropriate location of the neomeatus [21]. It has been documented that the penile inversion technique is associated with vaginal stenosis in approximately 10% of cases [21, 22]. Some authors emphasise the necessity for lifelong dilation of the neovagina, especially since the neovagina passes through a new opening through the pelvic floor muscles [21]. After peritoneal and intestinal vaginoplasty, a wide range of the TVL is reported: from 7.2 ± 1.5 cm at a mean follow-up of 43.8 months [8, 23, 24] to 14.7 cm at a mean follow-up of 29 months [10]. Ageing and absent dilation seem to have a high impact on stenosis and shortening of the neovagina in this collective [11]. As for this question, in our collective local estrogen therapy and dilation of the neovagina were offered and performed in symptomatic patients, yet we lack exact data concerning the effect on the prolapse. Although physiotherapy seems to influence the post-operative perception of pain after sex reassignment surgery in transfemale patients [25], studies assessing the effect of pelvic floor muscle training (PFMT) on genital prolapse in this collective are missing. The TVL and GH, along with other POP-Q parameters in the intestinal and peritoneal group, are subject to variation due to a wide range of operation techniques, indications, prior sex reassignment surgeries, and surgery-specific characteristics in these groups. There is a broad body of evidence indicating a correlation between the size of the GH and the severity of genital prolapse [26–29]. This suggests a potential protective effect of vaginal stenosis on its manifestation. Furthermore, the size of the GH has been shown to have a significant correlation with the symptoms of bulge, which appear to be more pronounced in cases of greater GH [30].

In conclusion, the penile inversion technique appears to be the optimal approach for addressing genital prolapse and TVL in the male-to-female transgender population. The results appear to be favourable in terms of anatomy, severity of future genital prolapse and sexual function. However, it is important to emphasise that it represents a first-line technique for patients without previous SRS. For instance, in cases of insufficient penile and scrotal skin, it is not appropriate. Other approaches, such as the peritoneal and intestinal repair will also gain more importance in the future, as the number of repeated and consecutive SRSs will increase with demographic changes and an ageing society. In addition, societal changes have led to a rise in the reported transgender population. As a result, the number of transgender surgeries will increase in the future. These developments will require and enable further prospective and randomised trials to assess therapeutic options concerning sex reassignment surgery and genital prolapse in transfemale patients.

## Conclusion

One in five transfemale patients who have undergone neovaginal construction as part of their SRS experience prolapse in the long term, with the highest rates observed in the peritoneal group. Both conservative and surgical interventions appear to significantly alleviate symptoms. These findings underscore the importance of awareness among patients and healthcare providers to ensure timely diagnosis and appropriate management.

## Data Availability

The raw data supporting the conclusion of this article will be made available by the corresponding author on request, because our data are confidential.
